# Tetra­kis(μ_2_-phenyl­acetato-κ^2^ *O*:*O*′)bis­[(isoquinoline-κ*N*)copper(II)]

**DOI:** 10.1107/S1600536809048697

**Published:** 2009-11-21

**Authors:** Meng-Jiao Li, Jing-Jing Nie, Duan-Jun Xu

**Affiliations:** a Department of Chemistry, Zhejiang University, People’s Republic of China

## Abstract

In the title centrosymmetric binuclear Cu^II^ complex, [Cu_2_(C_8_H_7_O_2_)_4_(C_9_H_7_N)_2_], the two Cu cations are bridged by four carboxyl­ate groups of the phenyl­acetate anions; each Cu cation is further coordinated by an isoquinoline ligand to complete the distorted CuO_4_N square-pyramidal geometry. The Cu cation is displaced by 0.2092 (8) Å from the basal plane formed by the four O atoms. Within the dinuclear mol­ecule, the Cu⋯Cu separation is 2.6453 (6) Å. Although a parallel, overlapped arrangement of isoquinoline ligands exists in the crystal structure; the longer face-to-face distance of 3.667 (5) Å suggests there is no π–π stacking between isoquinoline ring systems.

## Related literature

For general background to π–π stacking, see: Su & Xu (2004 ▶); Xu *et al.* (2007 ▶). For a related isoquinoline complex, see: Li *et al.* (2009 ▶). For Cu⋯Cu separations in multi-nuclear Cu^II^ complexes, see: Li *et al.* (2007 ▶, 2009 ▶).
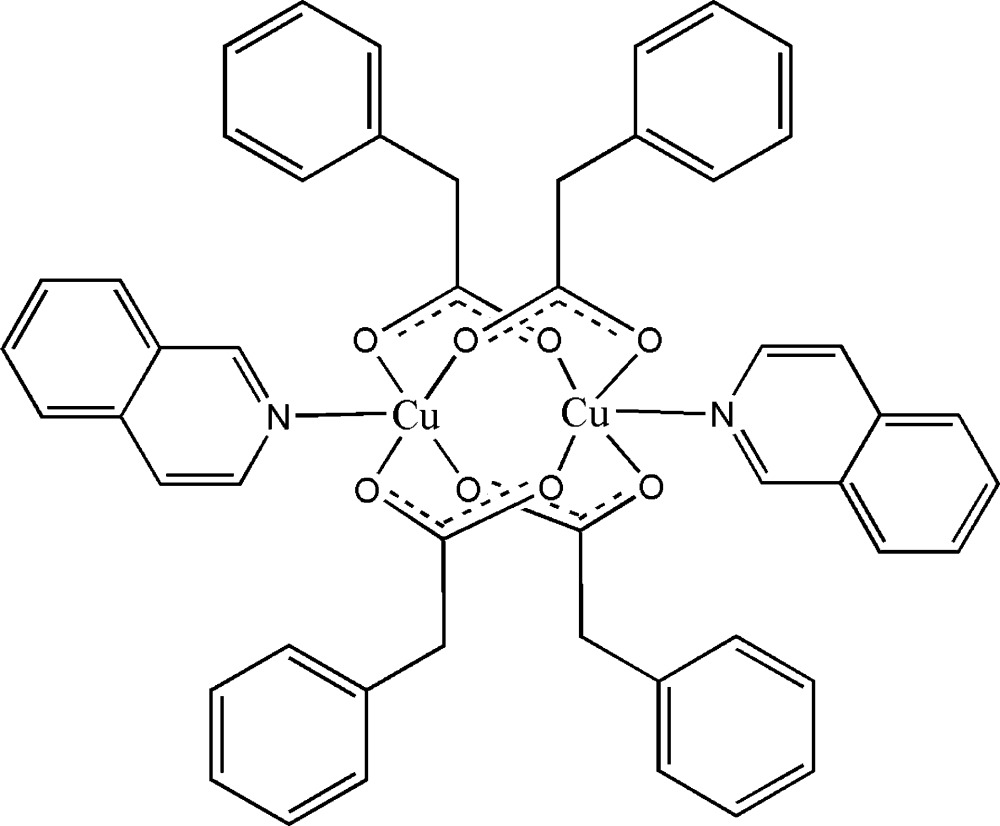


## Experimental

### 

#### Crystal data

[Cu_2_(C_8_H_7_O_2_)_4_(C_9_H_7_N)_2_]
*M* *_r_* = 925.94
Triclinic, 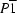

*a* = 8.2425 (15) Å
*b* = 11.251 (2) Å
*c* = 12.121 (2) Å
α = 94.594 (2)°
β = 90.178 (2)°
γ = 104.803 (4)°
*V* = 1082.9 (3) Å^3^
*Z* = 1
Mo *K*α radiation
μ = 1.04 mm^−1^
*T* = 294 K
0.26 × 0.22 × 0.16 mm

#### Data collection

Rigaku R-AXIS RAPID IP diffractometer
Absorption correction: multi-scan (*ABSCOR*; Higashi, 1995 ▶) *T*_min_ = 0.835, *T*_max_ = 0.920
11731 measured reflections
3837 independent reflections
3409 reflections with *I* > 2σ(*I*)
*R*_int_ = 0.025

#### Refinement

*R*[*F* ^2^ > 2σ(*F* ^2^)] = 0.032
*wR*(*F* ^2^) = 0.088
*S* = 1.09
3837 reflections
280 parameters
H-atom parameters constrained
Δρ_max_ = 0.29 e Å^−3^
Δρ_min_ = −0.20 e Å^−3^

### 

Data collection: *PROCESS-AUTO* (Rigaku, 1998 ▶); cell refinement: *PROCESS-AUTO*; data reduction: *CrystalStructure* (Rigaku/MSC, 2002 ▶); program(s) used to solve structure: *SIR92* (Altomare *et al.*, 1993 ▶); program(s) used to refine structure: *SHELXL97* (Sheldrick, 2008 ▶); molecular graphics: *ORTEP-3 for Windows* (Farrugia, 1997 ▶); software used to prepare material for publication: *WinGX* (Farrugia, 1999 ▶).

## Supplementary Material

Crystal structure: contains datablocks I, global. DOI: 10.1107/S1600536809048697/ng2685sup1.cif

Structure factors: contains datablocks I. DOI: 10.1107/S1600536809048697/ng2685Isup2.hkl

Additional supplementary materials:  crystallographic information; 3D view; checkCIF report

## Figures and Tables

**Table 1:** Selected bond lengths (Å)

Cu—O1	1.9786 (16)
Cu—O2^i^	1.9754 (16)
Cu—O3	1.9785 (17)
Cu—O4^i^	1.9761 (17)
Cu—N1	2.1522 (18)

Symmetry code: (i) 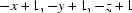.

**Fig. 1.**
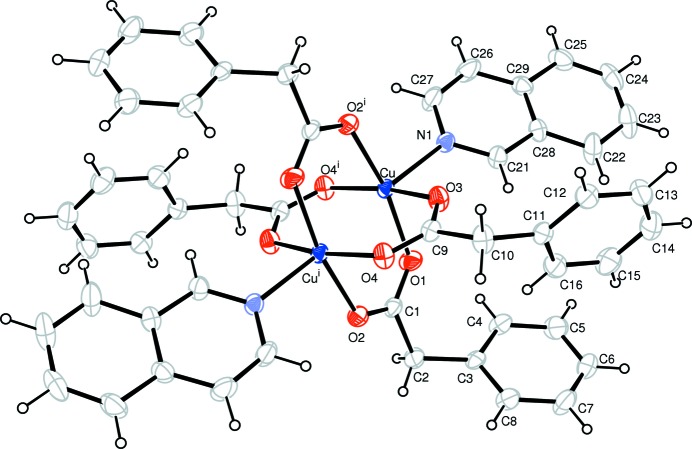
The molecular structure of the title compound with 30% probability displacement ellipsoids (arbitrary spheres for H atoms) [symmetry code: (i) 1 - x, 1 - y, 1 - z].

**Fig. 2.**
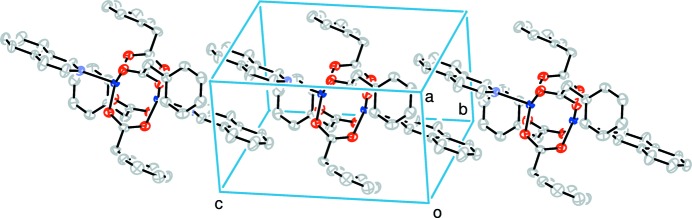
The unit cell packing diagram showing the parallel arrangement of isoquinoline ligands. H atoms have been omitted for clarity.

## supplementary crystallographic information

### Comment

As part of our ongoing investigation on the nature of π-π stacking (Su & Xu, 2004; Xu *et al.*, 2007), the title complex incorporating isoquinoline ligand has recently been prepared in the laboratory and its crystal structure is reported here.

The molecular structure is shown in Fig. 1. Four phenylacetate anions bridge two Cu^II^ cations to form the centro-symmetric complex. Within the dinuclear molecule the Cu···Cu separation of 2.6453 (6) Å is consistent with 2.646 Å found in a related binucealr Cu^II^ complex bridged by acetate anions (Li *et al.*, 2009) and 2.642 Å found in a polymeric Cu^II^ complex bridged by thiourea (Li *et al.* 2007). The Cu^II^ cation is coordinated by four carboxyl-O atoms from phenylacetate anions in the basal plane, an isoquinoline molecule further coordinates to the Cu^II^ cation in the apical position to complete the distorted square-pyramidal coordination geometry; the Cu^II^ cation is 0.2092 (8) Å deviated from the basal coordination plane.

The parallel, overlaped arrangement of isoquinoline ligands of adjacent complexes is observed in the crystal structure (Fig. 2). The face-to-face distance of 3.667 (5) Å suggests no π-π stacking between isoquinoline ring systems in the crystal structure.

### Experimental

Isoquinoline (0.23 ml, 2 mmol), copper dicholoride dihydrate (0.17 g, 1 mmol) and 2-phenylacetic acid (0.27 g, 2 mmol) were dissolved in ethanol (10 ml) at room temperature. The single crystals of the title compound were obtained from the solution after 2 d.

### Refinement

H atoms were placed in calculated positions with C—H = 0.93 (aromatic) and 0.97 Å (methylene) and refined in riding mode with *U*_iso_(H) = 1.2*U*_eq_(C).

### Crystal data

**Table:**

[Cu_2_(C_8_H_7_O_2_)_4_(C_9_H_7_N)_2_]	*Z* = 1
*M**_r_* = 925.94	*F*(000) = 478
Triclinic, *P*1	*D*_x_ = 1.420 Mg m^−^^3^
Hall symbol: -P 1	Mo *K*α radiation, λ = 0.71073 Å
*a* = 8.2425 (15) Å	Cell parameters from 5268 reflections
*b* = 11.251 (2) Å	θ = 2.0–25.0°
*c* = 12.121 (2) Å	µ = 1.04 mm^−^^1^
α = 94.594 (2)°	*T* = 294 K
β = 90.178 (2)°	Prism, blue
γ = 104.803 (4)°	0.26 × 0.22 × 0.16 mm
*V* = 1082.9 (3) Å^3^	

### Data collection

**Table:**

Rigaku R-AXIS RAPID IP diffractometer	3837 independent reflections
Radiation source: fine-focus sealed tube	3409 reflections with *I* > 2σ(*I*)
graphite	*R*_int_ = 0.025
Detector resolution: 10.0 pixels mm^-1^	θ_max_ = 25.2°, θ_min_ = 1.7°
ω scans	*h* = −9→9
Absorption correction: multi-scan (*ABSCOR*; Higashi, 1995)	*k* = −13→12
*T*_min_ = 0.835, *T*_max_ = 0.920	*l* = −14→14
11731 measured reflections	

### Refinement

**Table:**

Refinement on *F*^2^	Primary atom site location: structure-invariant direct methods
Least-squares matrix: full	Secondary atom site location: difference Fourier map
*R*[*F*^2^ > 2σ(*F*^2^)] = 0.032	Hydrogen site location: inferred from neighbouring sites
*wR*(*F*^2^) = 0.088	H-atom parameters constrained
*S* = 1.09	*w* = 1/[σ^2^(*F*_o_^2^) + (0.0476*P*)^2^ + 0.2372*P*] where *P* = (*F*_o_^2^ + 2*F*_c_^2^)/3
3837 reflections	(Δ/σ)_max_ < 0.001
280 parameters	Δρ_max_ = 0.28 e Å^−^^3^
0 restraints	Δρ_min_ = −0.20 e Å^−^^3^

### Special details

**Table:**

Geometry. All e.s.d.'s (except the e.s.d. in the dihedral angle between two l.s. planes) are estimated using the full covariance matrix. The cell e.s.d.'s are taken into account individually in the estimation of e.s.d.'s in distances, angles and torsion angles; correlations between e.s.d.'s in cell parameters are only used when they are defined by crystal symmetry. An approximate (isotropic) treatment of cell e.s.d.'s is used for estimating e.s.d.'s involving l.s. planes.
Refinement. Refinement of *F*^2^ against ALL reflections. The weighted *R*-factor *wR* and goodness of fit *S* are based on *F*^2^, conventional *R*-factors *R* are based on *F*, with *F* set to zero for negative *F*^2^. The threshold expression of *F*^2^ > σ(*F*^2^) is used only for calculating *R*-factors(gt) *etc*. and is not relevant to the choice of reflections for refinement. *R*-factors based on *F*^2^ are statistically about twice as large as those based on *F*, and *R*- factors based on ALL data will be even larger.

### Fractional atomic coordinates and isotropic or equivalent isotropic displacement parameters (Å^2^)

**Table:**

	*x*	*y*	*z*	*U*_iso_*/*U*_eq_	
Cu	0.44020 (3)	0.49777 (2)	0.39826 (2)	0.03660 (11)	
N1	0.3604 (2)	0.47759 (17)	0.22703 (15)	0.0433 (4)	
O1	0.3102 (2)	0.32991 (15)	0.42744 (15)	0.0580 (5)	
O2	0.4167 (2)	0.33169 (14)	0.59722 (14)	0.0526 (4)	
O3	0.6366 (2)	0.43173 (17)	0.36405 (14)	0.0546 (4)	
O4	0.7375 (2)	0.43275 (17)	0.53476 (14)	0.0522 (4)	
C1	0.3214 (3)	0.2823 (2)	0.5162 (2)	0.0447 (5)	
C2	0.2087 (4)	0.1536 (2)	0.5287 (2)	0.0581 (7)	
H2A	0.2451	0.1228	0.5945	0.070*	
H2B	0.0948	0.1603	0.5403	0.070*	
C3	0.2070 (3)	0.0608 (2)	0.4318 (2)	0.0446 (5)	
C4	0.1314 (3)	0.0679 (2)	0.3319 (2)	0.0582 (7)	
H4	0.0816	0.1321	0.3229	0.070*	
C5	0.1289 (4)	−0.0202 (3)	0.2444 (3)	0.0727 (9)	
H5	0.0783	−0.0143	0.1771	0.087*	
C6	0.2001 (5)	−0.1151 (3)	0.2565 (3)	0.0766 (9)	
H6	0.1963	−0.1746	0.1980	0.092*	
C7	0.2763 (4)	−0.1229 (3)	0.3536 (3)	0.0795 (9)	
H7	0.3270	−0.1868	0.3615	0.095*	
C8	0.2788 (4)	−0.0355 (2)	0.4415 (2)	0.0611 (7)	
H8	0.3302	−0.0422	0.5083	0.073*	
C9	0.7351 (3)	0.4067 (2)	0.4323 (2)	0.0436 (5)	
C10	0.8640 (3)	0.3418 (3)	0.3878 (2)	0.0595 (7)	
H10A	0.9673	0.4036	0.3764	0.071*	
H10B	0.8874	0.2907	0.4434	0.071*	
C11	0.8137 (3)	0.2622 (2)	0.2810 (2)	0.0457 (5)	
C12	0.9175 (3)	0.2738 (3)	0.1913 (2)	0.0567 (6)	
H12	1.0190	0.3339	0.1960	0.068*	
C13	0.8745 (4)	0.1989 (3)	0.0954 (2)	0.0694 (8)	
H13	0.9471	0.2084	0.0363	0.083*	
C14	0.7261 (4)	0.1106 (3)	0.0860 (3)	0.0748 (9)	
H14	0.6968	0.0602	0.0206	0.090*	
C15	0.6204 (4)	0.0969 (3)	0.1739 (3)	0.0755 (9)	
H15	0.5194	0.0363	0.1681	0.091*	
C16	0.6622 (3)	0.1718 (3)	0.2706 (2)	0.0611 (7)	
H16	0.5888	0.1620	0.3293	0.073*	
C21	0.3704 (3)	0.3795 (2)	0.1640 (2)	0.0500 (6)	
H21	0.4125	0.3205	0.1956	0.060*	
C22	0.3277 (5)	0.2486 (3)	−0.0130 (3)	0.0854 (10)	
H22	0.3657	0.1877	0.0185	0.103*	
C23	0.2778 (5)	0.2338 (4)	−0.1212 (3)	0.0947 (12)	
H23	0.2835	0.1626	−0.1640	0.114*	
C24	0.2186 (4)	0.3226 (4)	−0.1690 (2)	0.0805 (10)	
H24	0.1846	0.3101	−0.2432	0.097*	
C25	0.2095 (4)	0.4268 (3)	−0.1096 (2)	0.0751 (9)	
H25	0.1700	0.4859	−0.1429	0.090*	
C26	0.2524 (5)	0.5517 (3)	0.0722 (3)	0.0869 (11)	
H26	0.2134	0.6139	0.0437	0.104*	
C27	0.3021 (4)	0.5620 (3)	0.1800 (2)	0.0718 (9)	
H27	0.2950	0.6322	0.2238	0.086*	
C28	0.3213 (3)	0.3581 (2)	0.0513 (2)	0.0489 (6)	
C29	0.2602 (3)	0.4467 (2)	0.0038 (2)	0.0536 (6)	

### Atomic displacement parameters (Å^2^)

**Table:**

	*U*^11^	*U*^22^	*U*^33^	*U*^12^	*U*^13^	*U*^23^
Cu	0.03901 (17)	0.03174 (16)	0.03687 (17)	0.00689 (11)	−0.00402 (11)	−0.00272 (11)
N1	0.0469 (11)	0.0393 (10)	0.0415 (10)	0.0094 (8)	−0.0075 (8)	−0.0036 (8)
O1	0.0681 (12)	0.0368 (9)	0.0580 (11)	−0.0062 (8)	−0.0115 (9)	0.0023 (8)
O2	0.0614 (10)	0.0383 (9)	0.0497 (10)	−0.0013 (8)	−0.0008 (8)	0.0000 (7)
O3	0.0567 (10)	0.0683 (12)	0.0473 (9)	0.0337 (9)	−0.0039 (8)	−0.0028 (8)
O4	0.0468 (9)	0.0653 (11)	0.0480 (10)	0.0235 (8)	−0.0053 (7)	−0.0045 (8)
C1	0.0449 (13)	0.0322 (12)	0.0524 (14)	0.0036 (9)	0.0086 (11)	−0.0029 (10)
C2	0.0658 (17)	0.0384 (13)	0.0590 (16)	−0.0058 (12)	0.0118 (13)	−0.0005 (11)
C3	0.0421 (12)	0.0291 (11)	0.0561 (14)	−0.0025 (9)	0.0011 (10)	0.0031 (10)
C4	0.0609 (16)	0.0425 (14)	0.0711 (18)	0.0131 (12)	−0.0118 (13)	0.0048 (12)
C5	0.089 (2)	0.0604 (18)	0.0603 (18)	0.0047 (16)	−0.0197 (15)	−0.0001 (14)
C6	0.106 (3)	0.0479 (17)	0.071 (2)	0.0156 (16)	−0.0005 (18)	−0.0126 (14)
C7	0.099 (2)	0.0492 (17)	0.097 (3)	0.0351 (16)	−0.003 (2)	−0.0036 (16)
C8	0.0664 (17)	0.0503 (15)	0.0655 (17)	0.0126 (13)	−0.0117 (14)	0.0063 (13)
C9	0.0373 (12)	0.0417 (12)	0.0502 (14)	0.0092 (9)	−0.0029 (10)	−0.0028 (10)
C10	0.0443 (14)	0.0792 (19)	0.0599 (16)	0.0298 (13)	−0.0072 (12)	−0.0107 (14)
C11	0.0401 (12)	0.0482 (13)	0.0538 (14)	0.0212 (10)	0.0012 (10)	0.0025 (11)
C12	0.0448 (14)	0.0590 (16)	0.0663 (17)	0.0135 (12)	0.0083 (12)	0.0051 (13)
C13	0.076 (2)	0.079 (2)	0.0561 (17)	0.0257 (16)	0.0143 (14)	0.0014 (15)
C14	0.086 (2)	0.069 (2)	0.0677 (19)	0.0227 (17)	−0.0061 (17)	−0.0147 (16)
C15	0.0661 (19)	0.0560 (17)	0.094 (2)	0.0008 (14)	−0.0056 (17)	−0.0070 (16)
C16	0.0541 (16)	0.0623 (17)	0.0662 (17)	0.0135 (13)	0.0112 (13)	0.0056 (14)
C21	0.0524 (14)	0.0541 (15)	0.0463 (13)	0.0216 (11)	−0.0097 (11)	−0.0038 (11)
C22	0.105 (3)	0.097 (3)	0.0634 (19)	0.054 (2)	−0.0140 (17)	−0.0290 (18)
C23	0.104 (3)	0.122 (3)	0.059 (2)	0.044 (2)	−0.0057 (18)	−0.039 (2)
C24	0.079 (2)	0.115 (3)	0.0360 (15)	0.008 (2)	0.0016 (14)	−0.0062 (17)
C25	0.091 (2)	0.084 (2)	0.0436 (15)	0.0073 (17)	−0.0106 (14)	0.0131 (15)
C26	0.147 (3)	0.0507 (17)	0.070 (2)	0.0390 (19)	−0.039 (2)	0.0039 (15)
C27	0.116 (3)	0.0440 (15)	0.0592 (17)	0.0326 (16)	−0.0282 (16)	−0.0103 (13)
C28	0.0417 (13)	0.0595 (15)	0.0432 (13)	0.0121 (11)	−0.0005 (10)	−0.0062 (11)
C29	0.0570 (15)	0.0552 (15)	0.0424 (13)	0.0026 (12)	−0.0036 (11)	0.0066 (11)

### Geometric parameters (Å, °)

**Table:**

Cu—O1	1.9786 (16)	C10—C11	1.507 (3)
Cu—O2^i^	1.9754 (16)	C10—H10A	0.9700
Cu—O3	1.9785 (17)	C10—H10B	0.9700
Cu—O4^i^	1.9761 (17)	C11—C12	1.379 (3)
Cu—N1	2.1522 (18)	C11—C16	1.393 (3)
Cu—Cu^i^	2.6453 (6)	C12—C13	1.369 (4)
N1—C21	1.312 (3)	C12—H12	0.9300
N1—C27	1.333 (3)	C13—C14	1.362 (4)
O1—C1	1.253 (3)	C13—H13	0.9300
O2—C1	1.255 (3)	C14—C15	1.372 (4)
O2—Cu^i^	1.9754 (16)	C14—H14	0.9300
O3—C9	1.254 (3)	C15—C16	1.376 (4)
O4—C9	1.252 (3)	C15—H15	0.9300
O4—Cu^i^	1.9761 (17)	C16—H16	0.9300
C1—C2	1.527 (3)	C21—C28	1.407 (3)
C2—C3	1.506 (3)	C21—H21	0.9300
C2—H2A	0.9700	C22—C23	1.358 (5)
C2—H2B	0.9700	C22—C28	1.417 (4)
C3—C8	1.373 (4)	C22—H22	0.9300
C3—C4	1.378 (4)	C23—C24	1.383 (5)
C4—C5	1.388 (4)	C23—H23	0.9300
C4—H4	0.9300	C24—C25	1.345 (5)
C5—C6	1.360 (5)	C24—H24	0.9300
C5—H5	0.9300	C25—C29	1.419 (4)
C6—C7	1.352 (5)	C25—H25	0.9300
C6—H6	0.9300	C26—C27	1.356 (4)
C7—C8	1.387 (4)	C26—C29	1.403 (4)
C7—H7	0.9300	C26—H26	0.9300
C8—H8	0.9300	C27—H27	0.9300
C9—C10	1.513 (3)	C28—C29	1.388 (4)
			
O2^i^—Cu—O4^i^	87.53 (8)	C11—C10—C9	115.1 (2)
O2^i^—Cu—O1	167.83 (7)	C11—C10—H10A	108.5
O4^i^—Cu—O1	90.12 (8)	C9—C10—H10A	108.5
O2^i^—Cu—O3	90.58 (8)	C11—C10—H10B	108.5
O4^i^—Cu—O3	167.78 (7)	C9—C10—H10B	108.5
O1—Cu—O3	89.19 (8)	H10A—C10—H10B	107.5
O2^i^—Cu—N1	98.20 (7)	C12—C11—C16	117.7 (2)
O4^i^—Cu—N1	99.69 (7)	C12—C11—C10	121.4 (2)
O1—Cu—N1	93.96 (7)	C16—C11—C10	120.9 (2)
O3—Cu—N1	92.53 (7)	C13—C12—C11	121.5 (2)
O2^i^—Cu—Cu^i^	84.42 (5)	C13—C12—H12	119.2
O4^i^—Cu—Cu^i^	87.03 (5)	C11—C12—H12	119.2
O1—Cu—Cu^i^	83.53 (5)	C14—C13—C12	120.5 (3)
O3—Cu—Cu^i^	80.77 (5)	C14—C13—H13	119.8
N1—Cu—Cu^i^	172.85 (5)	C12—C13—H13	119.8
C21—N1—C27	117.3 (2)	C13—C14—C15	119.3 (3)
C21—N1—Cu	119.82 (16)	C13—C14—H14	120.3
C27—N1—Cu	122.90 (16)	C15—C14—H14	120.3
C1—O1—Cu	123.63 (14)	C14—C15—C16	120.7 (3)
C1—O2—Cu^i^	122.64 (16)	C14—C15—H15	119.6
C9—O3—Cu	126.83 (16)	C16—C15—H15	119.6
C9—O4—Cu^i^	119.64 (15)	C15—C16—C11	120.3 (3)
O1—C1—O2	125.7 (2)	C15—C16—H16	119.8
O1—C1—C2	117.9 (2)	C11—C16—H16	119.8
O2—C1—C2	116.4 (2)	N1—C21—C28	124.0 (2)
C3—C2—C1	114.8 (2)	N1—C21—H21	118.0
C3—C2—H2A	108.6	C28—C21—H21	118.0
C1—C2—H2A	108.6	C23—C22—C28	119.2 (3)
C3—C2—H2B	108.6	C23—C22—H22	120.4
C1—C2—H2B	108.6	C28—C22—H22	120.4
H2A—C2—H2B	107.5	C22—C23—C24	121.2 (3)
C8—C3—C4	117.9 (2)	C22—C23—H23	119.4
C8—C3—C2	120.5 (2)	C24—C23—H23	119.4
C4—C3—C2	121.6 (2)	C25—C24—C23	120.8 (3)
C3—C4—C5	120.4 (3)	C25—C24—H24	119.6
C3—C4—H4	119.8	C23—C24—H24	119.6
C5—C4—H4	119.8	C24—C25—C29	120.2 (3)
C6—C5—C4	120.5 (3)	C24—C25—H25	119.9
C6—C5—H5	119.8	C29—C25—H25	119.9
C4—C5—H5	119.8	C27—C26—C29	119.7 (3)
C7—C6—C5	119.9 (3)	C27—C26—H26	120.2
C7—C6—H6	120.0	C29—C26—H26	120.2
C5—C6—H6	120.0	N1—C27—C26	123.9 (3)
C6—C7—C8	119.9 (3)	N1—C27—H27	118.1
C6—C7—H7	120.0	C26—C27—H27	118.1
C8—C7—H7	120.0	C29—C28—C21	118.0 (2)
C3—C8—C7	121.3 (3)	C29—C28—C22	119.6 (2)
C3—C8—H8	119.3	C21—C28—C22	122.3 (3)
C7—C8—H8	119.3	C28—C29—C26	117.2 (2)
O4—C9—O3	125.3 (2)	C28—C29—C25	118.9 (3)
O4—C9—C10	116.9 (2)	C26—C29—C25	123.9 (3)
O3—C9—C10	117.8 (2)		

Symmetry codes: (i) −*x*+1, −*y*+1, −*z*+1.
